# Supplementary material for: Comprehensive Annotation and Functional Exploration of MicroRNAs in Lettuce
Source: Front Plant Sci. 2021 Dec 24;12:781836. doi: 10.3389/fpls.2021.781836 (PMC8739914; doi:10.3389/fpls.2021.781836)
Supplement: Supplementary file 2 [file Data_Sheet_8.ZIP › GSM2230748.plot/Lsa-miR394c_Lsat_1_v5_gn_1_87200.1_937_TPlot.pdf]

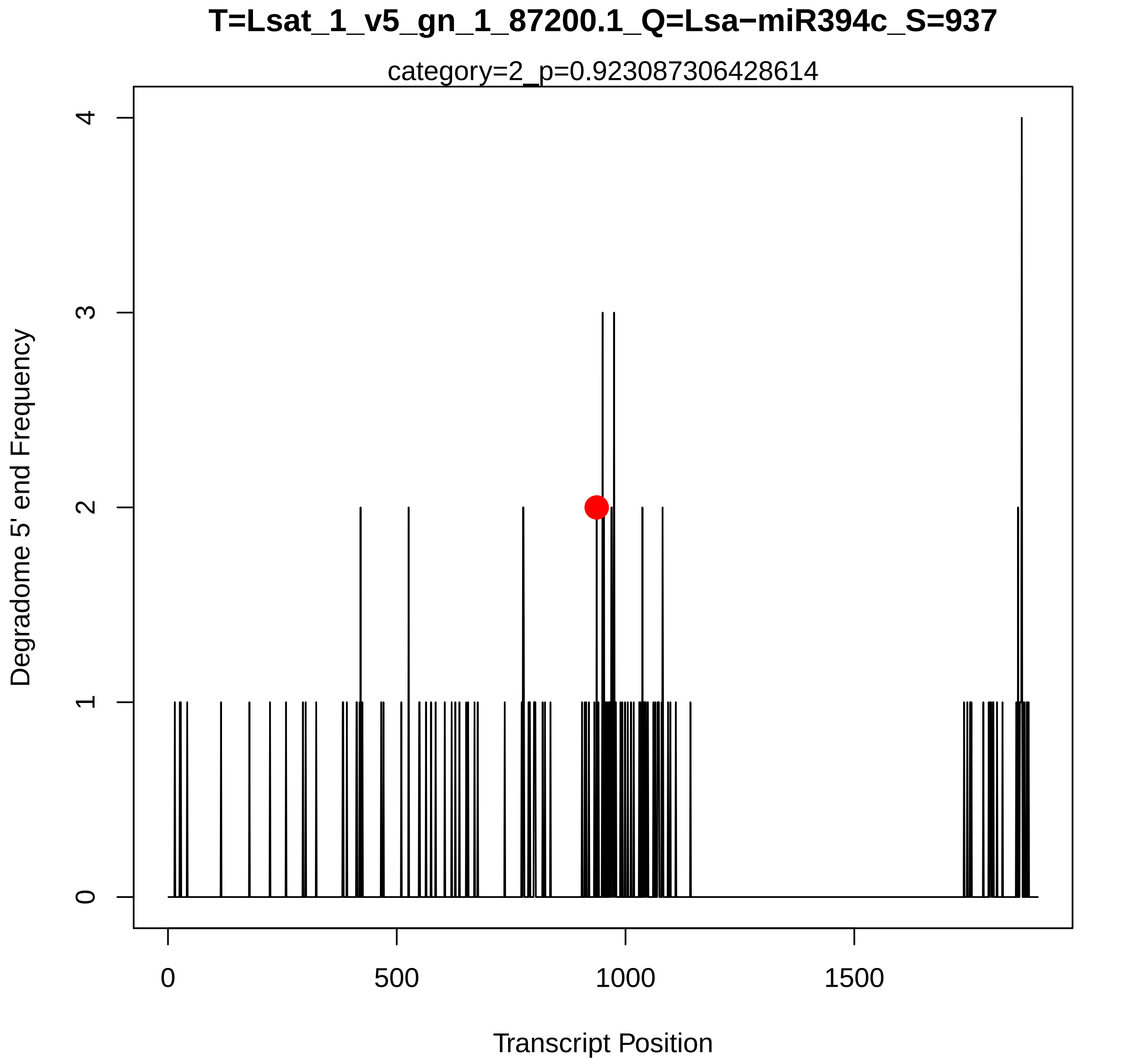

T=Lsat_1_v5_gn_1_87200.1_Q=Lsa−miR394c_S=937
category=2_p=0.923087306428614
Degradome 5' end Frequency
0
1
2
3
4
0
500
1000
1500
Transcript Position
